# Supplementary material for: S6K1-mediated phosphorylation of PDK1 impairs AKT kinase activity and oncogenic functions
Source: Nat Commun. 2022 Mar 22;13:1548. doi: 10.1038/s41467-022-28910-8 (PMC8941131; doi:10.1038/s41467-022-28910-8)
Supplement: Supplementary file 1 — Supplementary Information [file 41467_2022_28910_MOESM1_ESM.pdf]

Supplementary information for

S6K1-mediated phosphorylation of PDK1 impairs AKT kinase activity and oncogenic functions

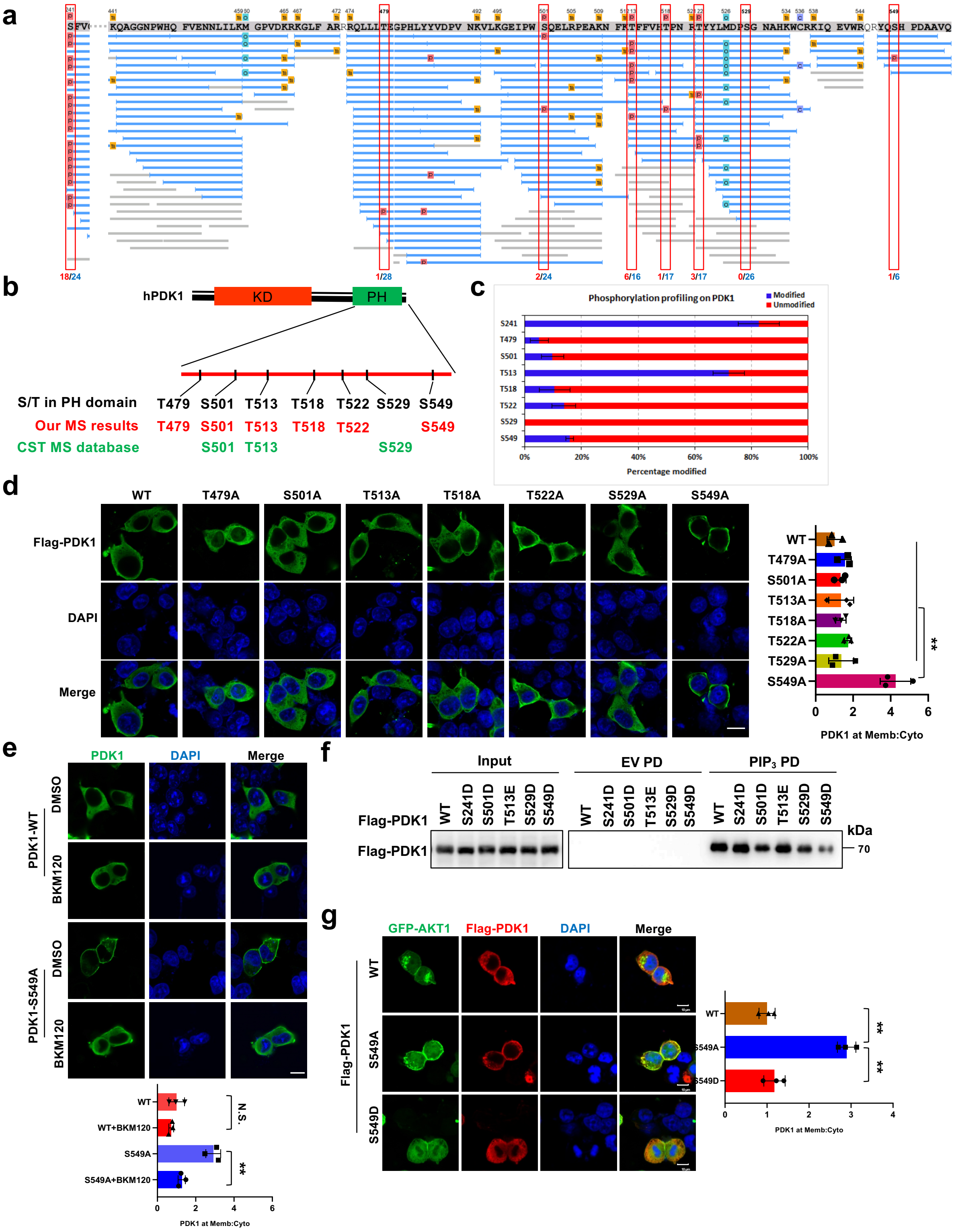
a
18/24
1/28
2/24
6/16
1/17
3/17
0/26
1/6
b
hPDK1
KD
PH
S/T in PH domain
T479 S501 T513 T518 T522 S529 S549
Our MS results
T479 S501 T513 T518 T522 S549
CST MS database
S501 T513 S529
c
Phosphorylation profiling on PDK1
Modified
Unmodified
S241
T479
S501
T513
T518
T522
S529
S549
0% 20% 40% 60% 80% 100%
Percentage modified
d
WT T479A S501A T513A T518A T522A S529A S549A
Flag-PDK1
DAPI
Merge
WT
T479A
S501A
T513A
T518A
T522A
T529A
S549A
0 2 4 6
PDK1 at Memb:Cyto
**
e
PDK1 DAPI Merge
PDK1-WT
DMSO
BKM120
PDK1-S549A
DMSO
BKM120
WT
WT+BKM120
S549A
S549A+BKM120
0 2 4 6
PDK1 at Memb:Cyto
N.S.
**
f
Input
EV PD
PIP3 PD
Flag-PDK1
WT S241D S501D T513E S529D S549D
WT S241D S501D T513E S529D S549D
WT S241D S501D T513E S529D S549D
kDa
Flag-PDK1
70
g
GFP-AKT1 Flag-PDK1 DAPI Merge
Flag-PDK1
WT
S549A
S549D
WT
S549A
S549D
0 1 2 3 4
PDK1 at Memb:Cyto
**
**

Supplementary Fig. 1 Potential phosphorylation sites in PDK1 PH domain. (a) Mass spectrometry analysis of PDK1 phosphorylation was performed with implicated Flag-PDK1 from HEK293T cells. The peptides with/without phosphorylation residues within the PH domain were summarized. The serine/threonine residues with phosphorylation modification were labeled red. **(b)** A schematic presentation of potential phosphorylation residues in the PDK1 PH domain by mass spectrometry (MS) approaches, coupled with the data mining derived from the CST MS database (https://www.phosphosite.org). **(c)** Phosphorylation sites on PDK1 were calculated. **(d)** IF staining of 293T cells transfected with different mutant forms of PDK1, scale bar, 10 μm . Mean PDK1 fluorescence intensity at plasma membrane relative cytosol was determined, data represent mean±SD, P=0.029, 0.049, 0.024, 0.015, 0.043, 0.048, 0.049. Greater than 60 cells were analyzed from 3 independent experiments. **(e)** IF staining of 293T cells transfected with different mutant forms of PDK1. Where indicated, the PI3K inhibitor (BKM120, 10 μM) was added for 1 hr, scale bar, 10 μm . Mean PDK1 fluorescence intensity at plasma membrane relative cytosol was determined, data represent mean±SD, P=0.325, 0.003. Greater than 60 cells were analyzed from 3 independent experiments. **(f)** IB analysis of PIP_3 pull-down products and WCL derived from 293T cells transfected with indicated constructs. **(g)** IF staining of 293T cells transfected with indicated constructs, scale bar, 10 μm. Mean PDK1 fluorescence intensity at plasma membrane relative cytosol was determined, data represent mean±SD, P=0.0003, 0.0009. Greater than 60 cells were analyzed from 3 independent experiments. Similar results were obtained in n≥3 independent experiments in **f**. Statistical significance was determined by two-tailed Student's *t*-test in **d, e, g**. N.S > 0.05, *P < 0.05, **P < 0.01. Source Data are provided in Source Data files. WT, wild type. PD, pulldown. Cyto, cytoplasm. Memb, membrane.

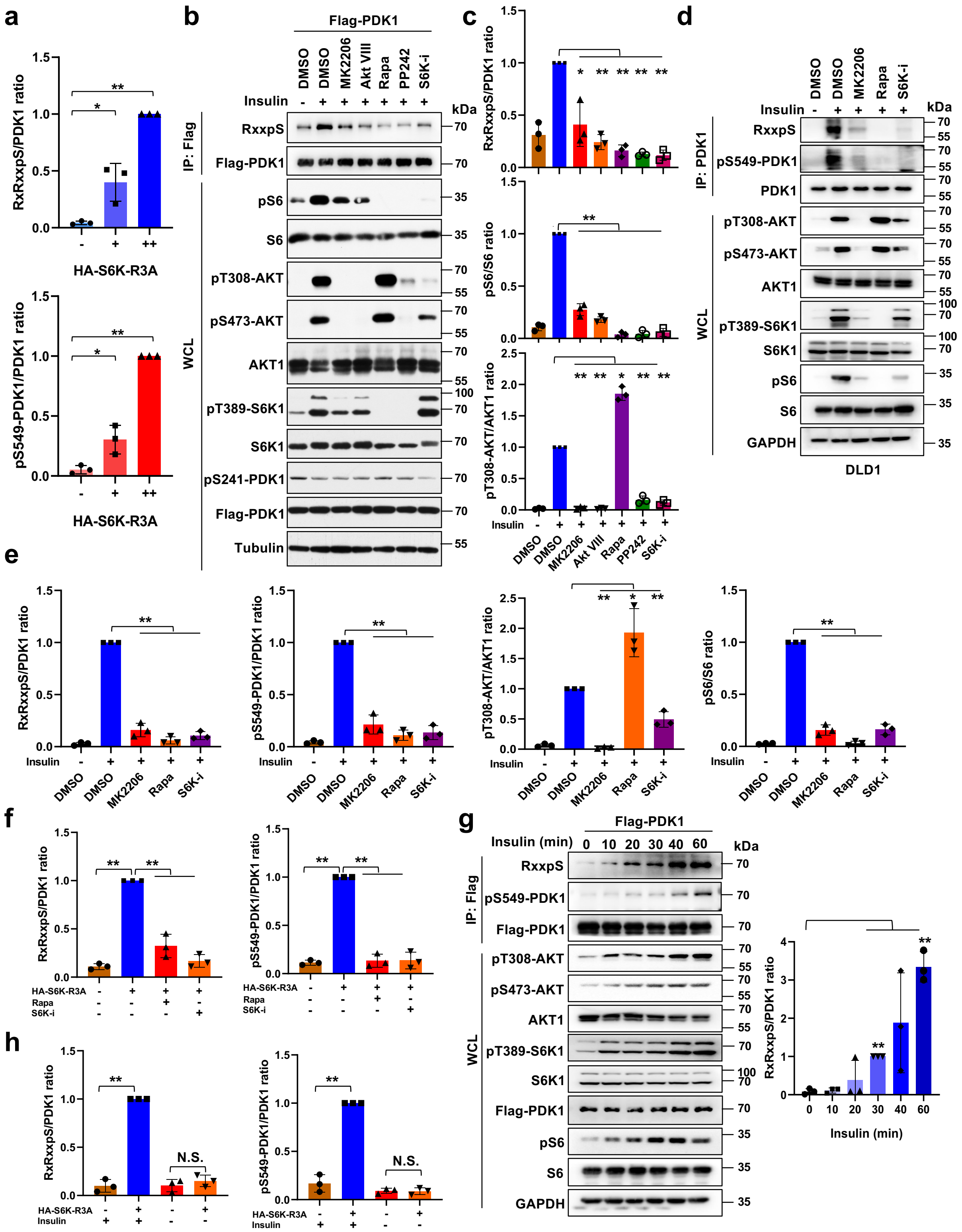

a
RxRxxpS/PDK1 ratio
HA-S6K-R3A
pS549-PDK1/PDK1 ratio
b
Flag-PDK1
DMSO
DMSO
MK2206
Akt VIII
Rapa
PP242
S6K-i
Insulin
kDa
IP: Flag
RxxpS
Flag-PDK1
WCL
pS6
S6
pT308-AKT
pS473-AKT
AKT1
pT389-S6K1
S6K1
pS241-PDK1
Tubulin
c
RxRxxpS/PDK1 ratio
pS6/S6 ratio
pT308-AKT/AKT1 ratio
d
IP: PDK1
RxxpS
pS549-PDK1
PDK1
WCL
pT308-AKT
pS473-AKT
AKT1
pT389-S6K1
S6K1
pS6
S6
GAPDH
DLD1
e
RxRxxpS/PDK1 ratio
pS549-PDK1/PDK1 ratio
pT308-AKT/AKT1 ratio
pS6/S6 ratio
f
RxRxxpS/PDK1 ratio
pS549-PDK1/PDK1 ratio
HA-S6K-R3A
Rapa
S6K-i
g
Flag-PDK1
Insulin (min)
IP: Flag
RxxpS
pS549-PDK1
Flag-PDK1
WCL
pT308-AKT
pS473-AKT
AKT1
pT389-S6K1
S6K1
Flag-PDK1
pS6
S6
GAPDH
RxRxxpS/PDK1 ratio
Insulin (min)
h
RxRxxpS/PDK1 ratio
pS549-PDK1/PDK1 ratio
N.S.
HA-S6K-R3A
Insulin

Supplementary Fig. 2 S6K1 phosphorylates PDK1 on Ser549. (a) Indicated protein in Fig 1d were quantified. (mean±SD, n=3, P=0.020, 4.33E-08, 0.026, 1.25E-06). **(b)** IB analysis of WCL and IP products derived from 293T cells transfected with Flag-PDK1 that were serum-starved for 24 hrs and then collected after insulin (100 nM) stimulation for 30 min. Where indicated, the kinase inhibitors (MK2206, 10 μM; AktVIII, 10 μM; Rapamycin, 20 nM; PP242, 1μM; S6K1-i, 10 μM) were added. Dimethylsulphoxide (DMSO) was used as a negative control. **(c)** Indicated protein in **(b)** were quantified. (mean±SD, n=3), *P < 0.05, **P < 0.01. **(d)** DLD1 cells were serum-starved for 24 hrs and then collected after treated with MK2206 (10 μM), Rapamycin (20 nM), S6K1-I (10 μM), for 1 hr before stimulated with insulin (100 nM) for 30 min. The resulting cells were subjected for IP and IB analysis, **(e)** Indicated protein in Fig 1d were quantified. (mean±SD, n=3), *P < 0.05, **P < 0.01. **(f)** Indicated protein in Fig 1e were quantified. (mean±SD, n=3), *P < 0.05, **P < 0.01. **(g)** IB analysis of WCL derived from 293T cells transfected with Flag-PDK1 that were serum-starved for 12 h and then treated with insulin (100 nM) for the indicated time periods before collection for IB analysis. Indicated protein were quantified. (mean±SD, n=3, P=2.95E-05, 0.076, 0.00015). **(h)** Indicated protein in Fig 1f were quantified. (mean±SD, n=3, P=1.85E-05, 0.41, 8.91E-05, 0.90). Statistical significance was determined by two-tailed Student's *t*-test in **a, c, e, f, g, h.** N.S > 0.05, *P < 0.05, **P < 0.01. Source Data are provided in Source Data files. WCL, whole cell lysate. IP, immunoprecipitation. WT, wild type.

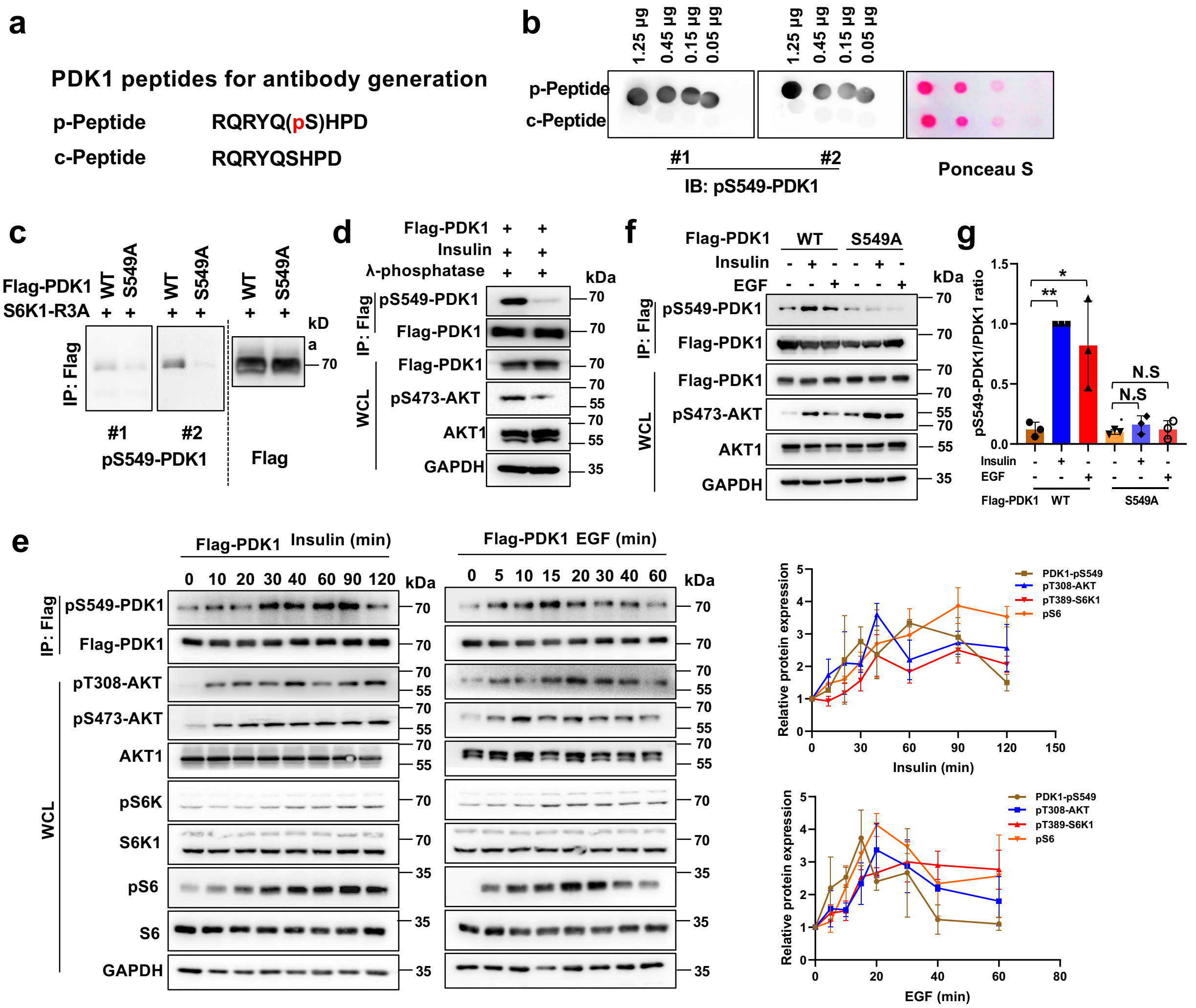
a
PDK1 peptides for antibody generation
p-Peptide RQRYQ(pS)HPD
c-Peptide RQRYQSHPD
b
1.25 μg
0.45 μg
0.15 μg
0.05 μg
p-Peptide
c-Peptide
#1
#2
IB: pS549-PDK1
Ponceau S
c
Flag-PDK1
S6K1-R3A
WT
S549A
IP: Flag
#1
#2
pS549-PDK1
Flag
kDa
70
d
Flag-PDK1
Insulin
λ-phosphatase
IP: Flag
pS549-PDK1
Flag-PDK1
WCL
pS473-AKT
AKT1
GAPDH
f
Flag-PDK1
WT
S549A
Insulin
EGF
g
pS549-PDK1/PDK1 ratio
N.S
e
Flag-PDK1 Insulin (min)
Flag-PDK1 EGF (min)
pT308-AKT
pS6K
S6K1
pS6
S6
PDK1-pS549
pT389-S6K1
Relative protein expression
Insulin (min)
EGF (min)

Supplementary Fig. 3 Validation of the anti-pS549-PDK1 antibody. (a) The amino sequence of PDK1 peptides for antibody generation. **(b)** The indicated synthetic peptides were dotted on nitrocellulose membrane for IB analysis. **(c)** IB analysis of WCL and IP products derived from 293T cells transfected with indicated constructs. **(d)** IB analysis of WCL and IP products derived from 293T cells transfected with Flag-PDK1 and treated with/without λ-phosphatase that were serum-starved for 12 hrs and then collected after insulin (100 nM) stimulation for 30 min. **(e)** IB analysis of WCL derived from 293T cells transfected with Flag-PDK1 that were serum-starved for 12 h and then treated with insulin (100 nM) or EGF (10 ng/ml) for the indicated time periods before collection for IB analysis (top panels), which the time curve of indicated protein were further monitored (bottom panels). (mean±SD, n=3) **(f)** IB analysis of WCL and IP products derived from 293T cells transfected with Flag-PDK1 (WT, S549A) that were serum-starved for 12 hrs and then collected after insulin (100 nM) or EGF (10 ng/ml) stimulation for 30 min. **(g)** Indicated proteins in **(f)** were quantified. (mean±SD, n=3, P=0.002, 0.03). Statistical significance was determined by two-tailed Student's *t*-test in **g**. N.S > 0.05, *P < 0.05, **P < 0.01. Source Data are provided in Source Data files. WCL, whole cell lysate. IP, immunoprecipitation. WT, wild type.

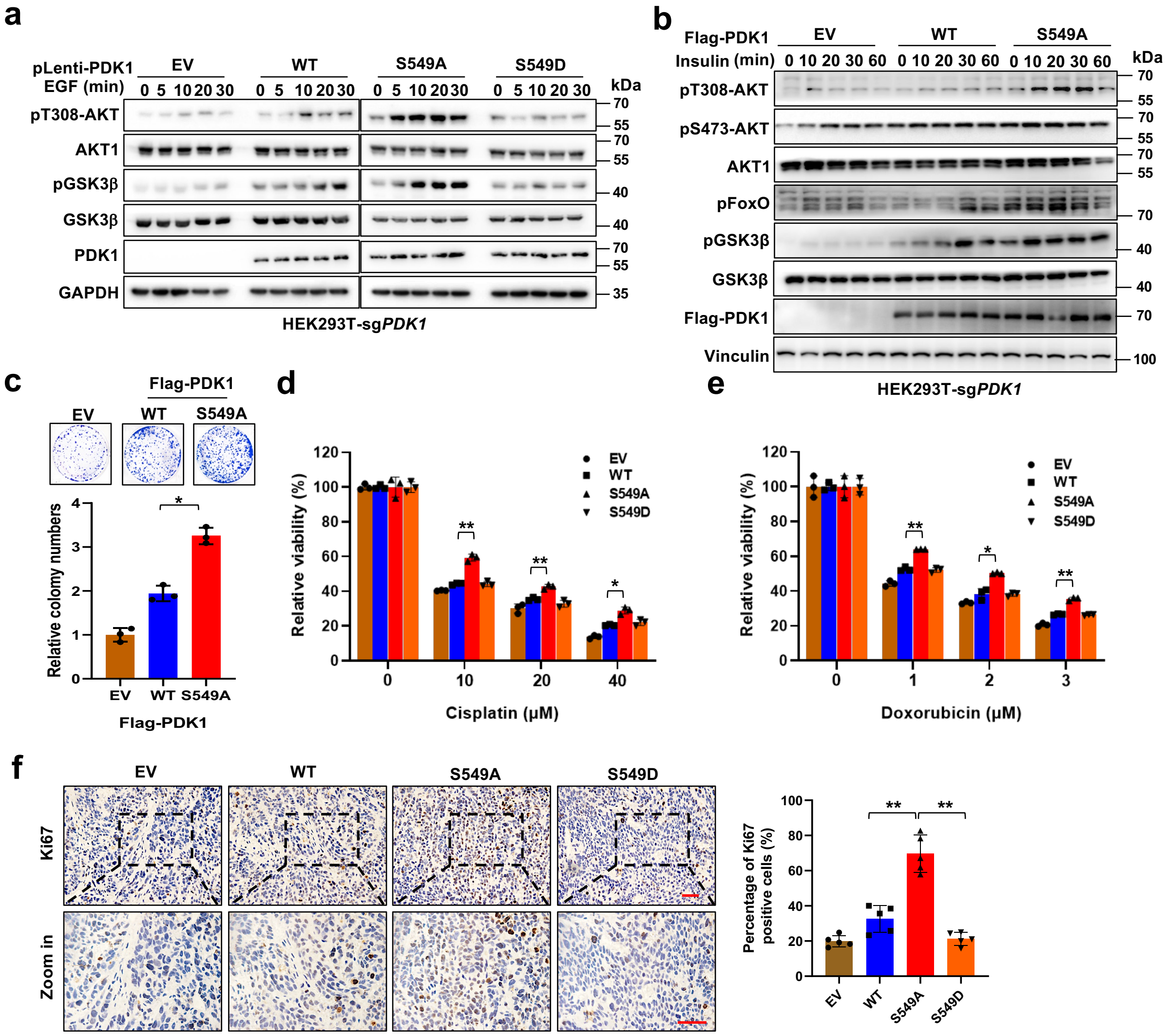
a
pLenti-PDK1
EV
WT
S549A
S549D
EGF (min)
0 5 10 20 30
kDa
pT308-AKT
AKT1
pGSK3β
GSK3β
PDK1
GAPDH
70
55
40
35
HEK293T-sgPDK1
b
Flag-PDK1
Insulin (min)
0 10 20 30 60
pT308-AKT
pS473-AKT
AKT1
pFoxO
pGSK3β
GSK3β
Flag-PDK1
Vinculin
100
HEK293T-sgPDK1
c
Flag-PDK1
EV
WT
S549A
Relative colony numbers
Flag-PDK1
d
Relative viability (%)
Cisplatin (μM)
EV
WT
S549A
S549D
e
Relative viability (%)
Doxorubicin (μM)
f
EV
WT
S549A
S549D
Ki67
Zoom in
Percentage of Ki67 positive cells (%)

Supplementary Fig. 4 PDK1-S549 Phosphorylation inhibits AKT kinase activity and oncogenic functions. (a) IB analysis of WCL derived from CRISPR/CAS9 mediated 293T-PDK1 knockout cells transfected with the indicated Flag-PDK1 constructs that were serum-starved for 12 h and then treated with the EGF (10 μg/ml) for the indicated time periods before collection for IB analysis. **(b, c)** IB analysis of WCL derived from 293T-PDK1 knockout cells transfected with Flag-PDK1(WT, S549A) that were serum-starved for 12 h and then treated with insulin (100 nM) for the indicated time periods before collection for IB analysis. Resulting cells were subjected to colony formation assays (**c**, top panel). The relative colony numbers were normailized (**c**, bottom panel). (mean±SD, n=3, P=0.012). **(d, e)** Cells generated in (Fig. 2a) were cultured in 10% FBS-containing medium with the indicated concentrations of Cisplatin and Doxorubicin for 48 h before subjecting for the cell viability assays. (mean±SD, n=3), *P < 0.05; **P < 0.01. **(f)** Graphic representation of Ki67 staining of derived tumor tissues (top panels), which were further normalized and quantified (bottom panels). (mean±SD, n=5, P=0.008, 0.0002), scale bar, 50 μm. Statistical significance was determined by two-tailed Student's t-test in **c, d, e, f**. N.S > 0.05, *P < 0.05, **P < 0.01. Source Data are provided in Source Data files. EV, empty vector. WT, wild type.

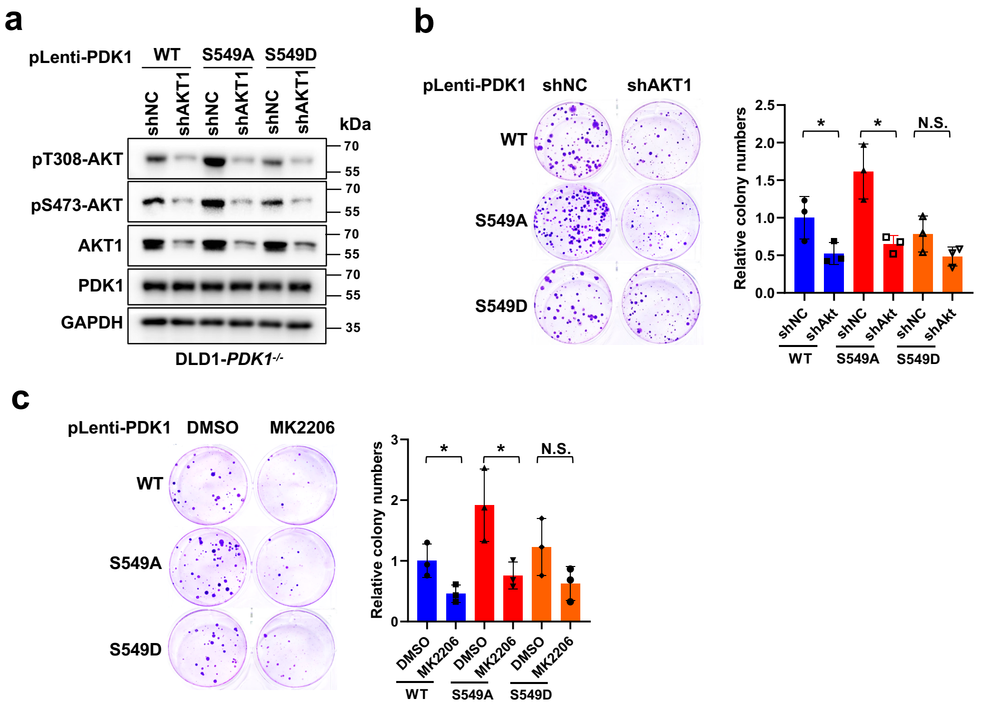

Supplementary Fig. 5 S549A-PDK1 oncogenic funcitons are depend on the AKT kinase. (a-b) DLD1-*PDK1* knockout cells stably expressed indicated constructs were infected with shSrc or shAKT1 lentivirus. Cells were selected with puromycin (1 μg/ml) for 72 hrs to eliminate uninfected cells and used for IB analyses **(a)**. Resulting cells were subjected to colony formation assays (b, left panel). The relative colony numbers were normailized (**b**, right panel). (mean±SD, n=3, P=0.029, 0.012, 0.127). **(c)** Cells generated in Fig. 2a were subjected for colony formation assays, where indicated, the MK2206 (10 μM) was added. The relative colony numbers were normailized (right panel). (mean±SD, n=3, P=0.039, 0.035, 0.128). Dimethylsulphoxide (DMSO) was used as a negative control. Statistical significance was determined by two-tailed Student's *t*-test in **b, c.** N.S > 0.05, *P < 0.05, **P < 0.01. Source Data are provided in Source Data files. WT, wild type.

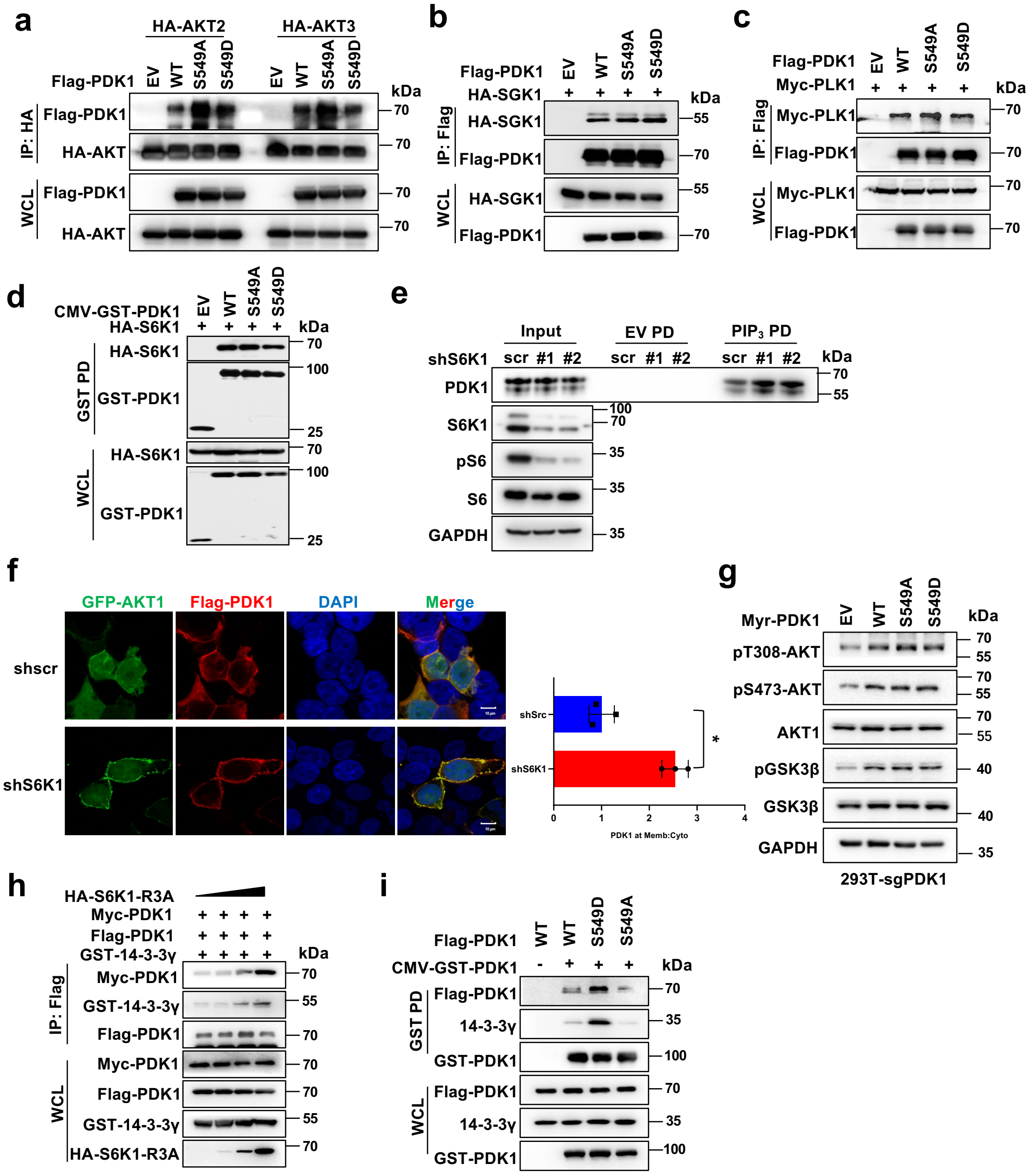

a
HA-AKT2
HA-AKT3
Flag-PDK1
EV
WT
S549A
S549D
kDa
IP: HA
Flag-PDK1
HA-AKT
WCL
70
b
HA-SGK1
IP: Flag
55
c
Myc-PLK1
d
CMV-GST-PDK1
HA-S6K1
GST PD
GST-PDK1
100
25
e
Input
EV PD
PIP3 PD
shS6K1
scr
#1
#2
PDK1
S6K1
pS6
S6
GAPDH
35
f
GFP-AKT1
DAPI
Merge
shscr
shS6K1
shSrc
PDK1 at Memb:Cyto
*
0
1
2
3
4
g
Myr-PDK1
pT308-AKT
pS473-AKT
AKT1
pGSK3β
GSK3β
40
293T-sgPDK1
h
HA-S6K1-R3A
Myc-PDK1
GST-14-3-3γ
i
14-3-3γ

Supplementary Fig. 6 Phosphorylation of PDK1 by S6K1 enhances its dimerization. (a-d) IB analysis of WCL and IP products derived from 293T cells transfected with indicated constructs. **(e)** IB analysis of PIP_3 pull-down products and WCL derived from 293T control or *S6K1* knockdown cells. **(f)** IF staining of 293T control or *S6K1* knockdown cells transfected with indicated constructs, scale bar, 10 μm. Mean PDK1 fluorescence intensity at plasma membrane relative cytosol was determined, data represent mean±SD, P=0.035. Greater than 60 cells were analyzed from 3 independent experiments. **(g-i)** IB analysis of WCL and IP products derived from 293T cells transfected with indicatd constructs. Similar results were obtained in n≥3 independent experiments in **a-e, g-i**. Statistical significance was determined by two-tailed Student's *t*-test in **f**. *P < 0.05. Source Data are provided in Source Data files. EV, empty vector. WCL, whole cell lysate. IP, immunoprecipitation. WT, wild type. PD, pulldown. Scr, scramble.

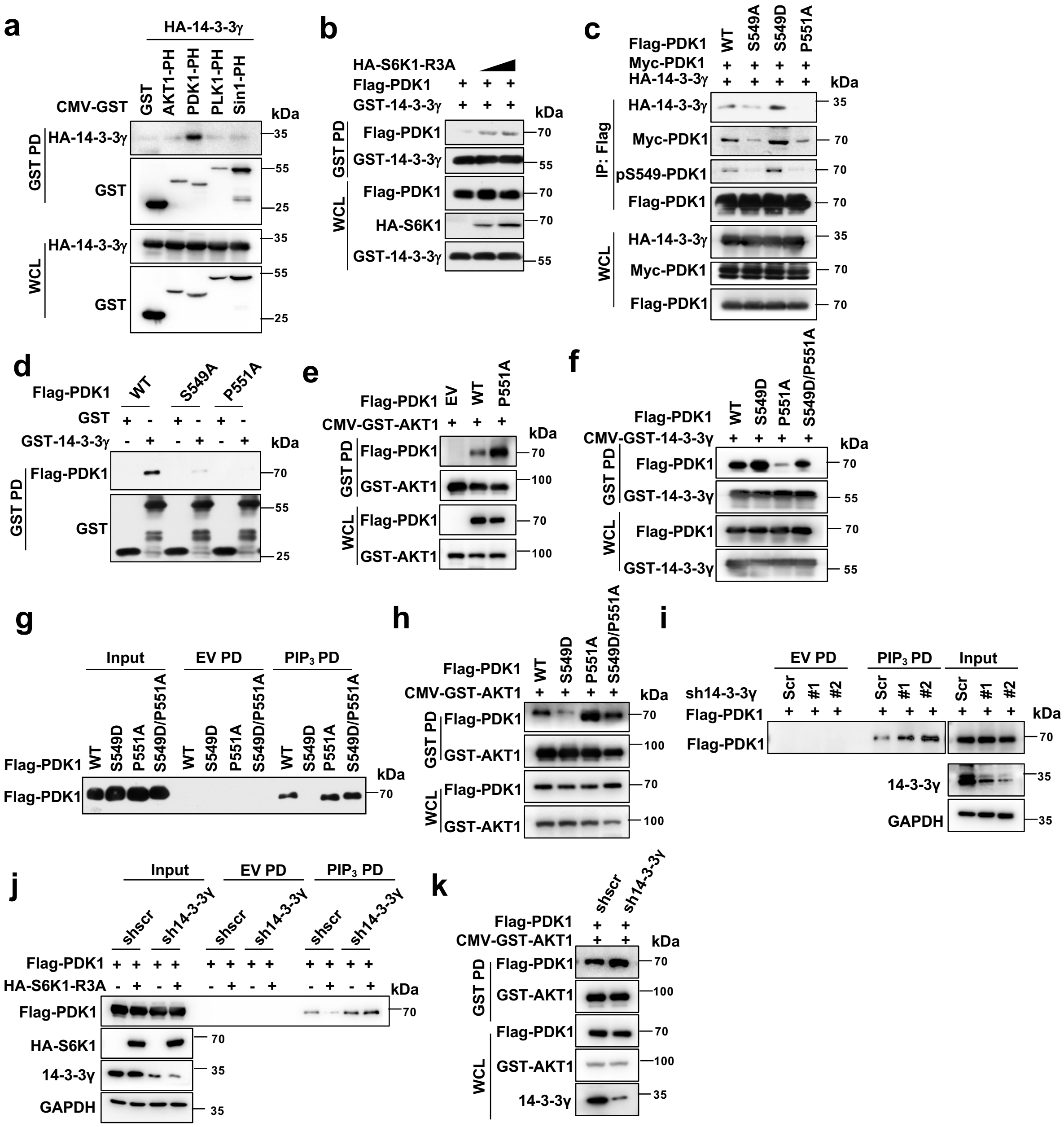

a
HA-14-3-3γ
CMV-GST
GST
AKT1-PH
PDK1-PH
PLK1-PH
Sin1-PH
kDa
GST PD
HA-14-3-3γ
GST
WCL
HA-14-3-3γ
GST
35
55
25
b
HA-S6K1-R3A
Flag-PDK1
GST-14-3-3γ
kDa
GST PD
Flag-PDK1
GST-14-3-3γ
WCL
Flag-PDK1
HA-S6K1
GST-14-3-3γ
70
55
c
Flag-PDK1
WT
S549A
S549D
P551A
Myc-PDK1
HA-14-3-3γ
kDa
IP: Flag
HA-14-3-3γ
Myc-PDK1
pS549-PDK1
Flag-PDK1
WCL
HA-14-3-3γ
Myc-PDK1
Flag-PDK1
35
70
d
Flag-PDK1
WT
S549A
P551A
GST
GST-14-3-3γ
kDa
GST PD
Flag-PDK1
GST
70
55
25
e
Flag-PDK1
EV
WT
P551A
CMV-GST-AKT1
kDa
GST PD
Flag-PDK1
GST-AKT1
WCL
Flag-PDK1
GST-AKT1
70
100
f
Flag-PDK1
WT
S549D
P551A
S549D/P551A
CMV-GST-14-3-3γ
kDa
GST PD
Flag-PDK1
GST-14-3-3γ
WCL
Flag-PDK1
GST-14-3-3γ
70
55
g
Input
EV PD
PIP3 PD
Flag-PDK1
WT
S549D
P551A
S549D/P551A
kDa
Flag-PDK1
70
h
Flag-PDK1
WT
S549D
P551A
S549D/P551A
CMV-GST-AKT1
kDa
GST PD
Flag-PDK1
GST-AKT1
WCL
Flag-PDK1
GST-AKT1
70
100
i
EV PD
PIP3 PD
Input
sh14-3-3γ
Scr
#1
#2
Flag-PDK1
kDa
Flag-PDK1
14-3-3γ
GAPDH
70
35
j
Input
EV PD
PIP3 PD
shscr
sh14-3-3γ
Flag-PDK1
HA-S6K1-R3A
kDa
Flag-PDK1
HA-S6K1
14-3-3γ
GAPDH
70
35
k
shscr
sh14-3-3γ
Flag-PDK1
CMV-GST-AKT1
kDa
GST PD
Flag-PDK1
GST-AKT1
WCL
Flag-PDK1
GST-AKT1
14-3-3γ
70
100
35

Supplementary Fig. 7 14-3-3 functions in modulating PDK1/AKT pathway. (a-f) IB analysis of WCL, IP and GST-pulldown products derived from 293T cells transfected with indicated constructs. **(g-h)** IB analysis of PIP_3 pulldown **(g)** or GST pulldown **(h)** products and WCL derived from 293T cells transfected with indicated constructs. **(i-j)** IB analysis of PIP_3 pulldown products and WCL derived from 293T control or *14-3-3γ* knockdown cells transfected with indicated constructs. **(k)** IB analysis of WCL and GST-pulldown derived from 293T control or *14-3-3γ* knockdown cells transfected with indicated constructs. Similar results were obtained in n≥3 independent experiments in **a-k**. Source Data are provided in Source Data files. EV, empty vector. WCL, whole cell lysate. IP, immunoprecipitation. WT, wild type. PD, pulldown.

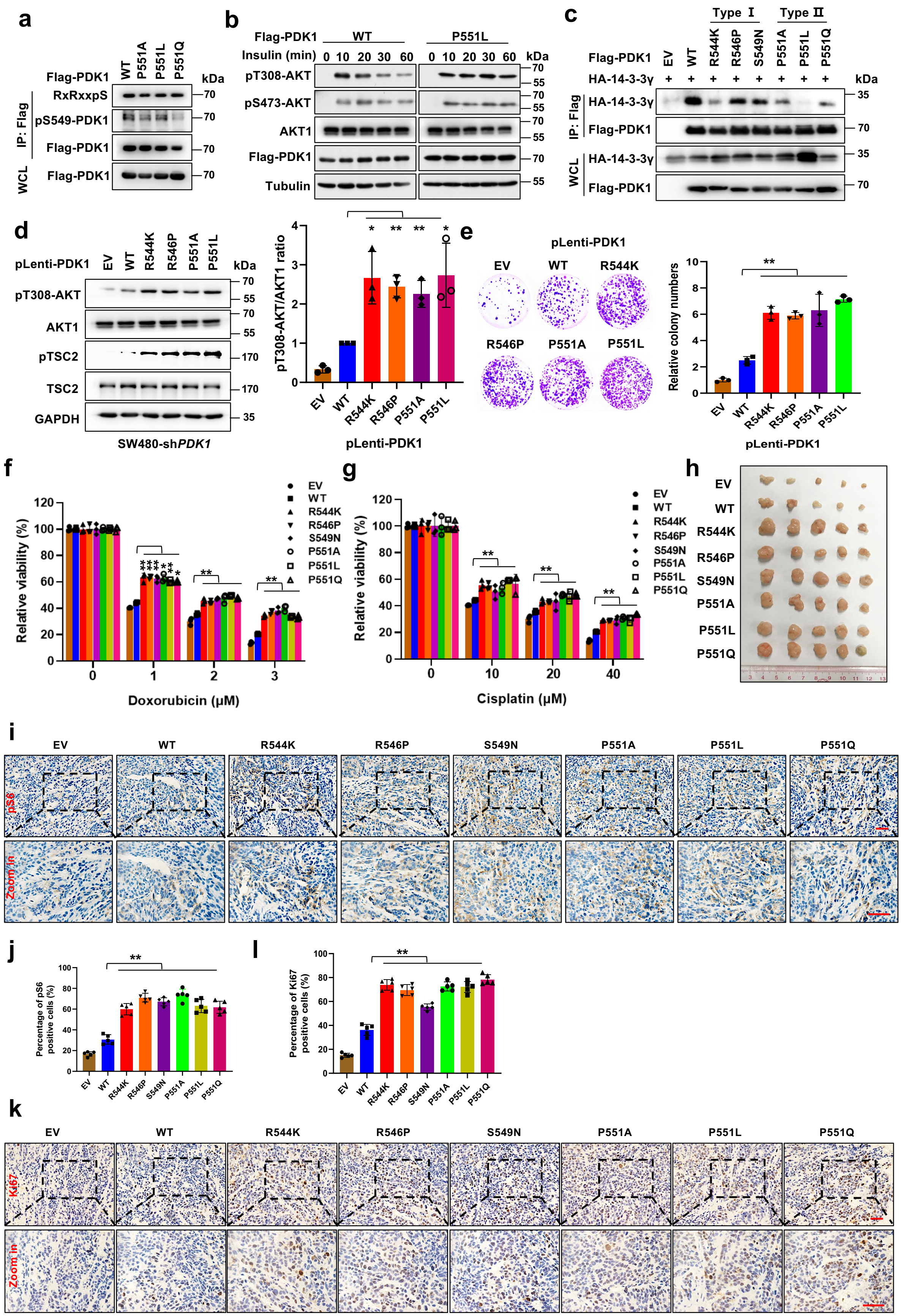
a
Flag-PDK1
WT
P551A
P551L
P551Q
kDa
IP: Flag
RxRxxpS
pS549-PDK1
Flag-PDK1
WCL
Flag-PDK1
b
Flag-PDK1
WT
P551L
Insulin (min)
0 10 20 30 60
pT308-AKT
pS473-AKT
AKT1
Flag-PDK1
Tubulin
c
Type I
Type II
EV
R544K
R546P
S549N
HA-14-3-3γ
d
pLenti-PDK1
pT308-AKT
pTSC2
TSC2
GAPDH
SW480-shPDK1
pT308-AKT/AKT1 ratio
e
Relative colony numbers
f
Relative viability (%)
Doxorubicin (μM)
g
Cisplatin (μM)
h
i
pS6
Zoom in
j
Percentage of pS6 positive cells (%)
k
Ki67
l
Percentage of Ki67 positive cells (%)

Supplementary Fig. 8 Patients associated PDK1 mutants promote tumor growth. (a) IB analysis of WCL and IP products derived from 293T cells transfected with indicated constructs. (**b**) IB analysis of WCL derived from 293T cells transfected with Flag-PDK1(WT, P551L) that were serum-starved for 12 h and then treated with the insulin (100 nM) for the indicated time periods before collection for IB analysis. **(c)** IB analysis of WCL and IP products derived from 293T cells transfected with HA-14-3-3γ and indicated constructs. **(d)** IB analysis of SW480-PDK1 knockdown cells stably infected with indicated constructs. (mean±SD, n=3), *P < 0.05, **P < 0.01. (**e**) Cells generated in A were subjected to colony formation. (mean±SD, n=3), **P < 0.01. **(f, g)** Cells generated in (Fig. 6a) were cultured in 10% FBS-containing medium with the indicated concentrations of Cisplatin and Doxorubicin for 48 h before subjecting for the cell viability assays. (mean±SD, n=3), *P < 0.05, **P < 0.01. **(h)** Cells generated in (Fig. 6a) were subjected to mouse xenograft assays, n=5 mice. **(i-l)** Representative immunohistochemistry images of Ki67 and pS6 IHC staining derived from tumor tissues (**i,k**) and quantified in **(j, P=0.001, 5.66E-06, 0.0003, 2.81E-05, 0.0025, 0.0009, l, P=0.0001, 0.0001, 0.002, 4.05E-06, 0.0005, 6.54E-06)**, (mean±SD, n=5). Scale bar, 50 µm. Similar results were obtained in n ⩾3 independent experiments in **a-c**. Statistical significance was determined by two-tailed Student's *t*-test in **d, e, f, g, j, l**. *P < 0.05, **P < 0.01. Source Data are provided in Source Data files. EV, empty vector. WCL, whole cell lysate. WT, wild type.

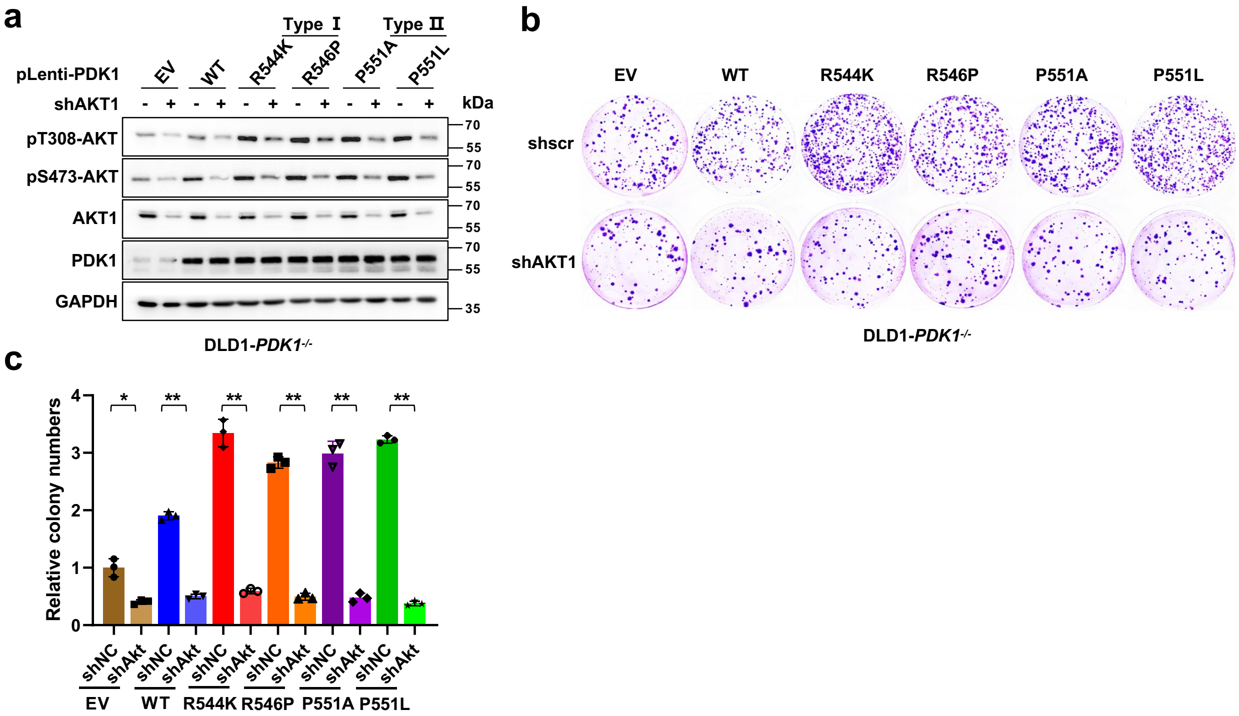

Supplementary Fig. 9 Mutations of PDK1 promote tumor growth via activating AKT kinase. (a-b) DLD1-*PDK1* knockout cells stably expressed indicated constructs were infected with shSrc or shAKT1 lentivirus. Cells were selected with puromycin (1 μg/ml) for 72 hrs to eliminate uninfected cells and used for IB analyses **(a)**. Resulting cells were subjected to colony formation assays **(b)**. **(c)** The relative colony numbers were normailized. (mean±SD, n=3, P=0.032, 0.0005, 0.002, 0.0008, 0.001, 0.0002), *P < 0.05, **P < 0.01. Statistical significance was determined by two-tailed Student's *t*-test in **c**. Source Data are provided in Source Data files.
