# Supplementary material for: S6K1-mediated phosphorylation of PDK1 impairs AKT kinase activity and oncogenic functions
Source: Nat Commun. 2022 Mar 22;13:1548. doi: 10.1038/s41467-022-28910-8 (PMC8941131; doi:10.1038/s41467-022-28910-8)
Supplement: Supplementary file 3 — Reporting Summary [file 41467_2022_28910_MOESM3_ESM.pdf]

nature research

Corresponding author(s): Jianping guo

Last updated by author(s): Feb 1, 2022

Reporting Summary

Nature Research wishes to improve the reproducibility of the work that we publish. This form provides structure for consistency and transparency in reporting. For further information on Nature Research policies, see Authors & Referees and the Editorial Policy Checklist.

Statistics

For all statistical analyses, confirm that the following items are present in the figure legend, table legend, main text, or Methods section.

n/a	Confirmed	
☐	☒	The exact sample size (*n*) for each experimental group/condition, given as a discrete number and unit of measurement
☐	☒	A statement on whether measurements were taken from distinct samples or whether the same sample was measured repeatedly
☐	☒	The statistical test(s) used AND whether they are one- or two-sided<br>*Only common tests should be described solely by name; describe more complex techniques in the Methods section.*
☒	☐	A description of all covariates tested
☒	☐	A description of any assumptions or corrections, such as tests of normality and adjustment for multiple comparisons
☐	☒	A full description of the statistical parameters including central tendency (e.g. means) or other basic estimates (e.g. regression coefficient) AND variation (e.g. standard deviation) or associated estimates of uncertainty (e.g. confidence intervals)
☐	☒	For null hypothesis testing, the test statistic (e.g. *F*, *t*, *r*) with confidence intervals, effect sizes, degrees of freedom and *P* value noted<br>*Give P values as exact values whenever suitable.*
☒	☐	For Bayesian analysis, information on the choice of priors and Markov chain Monte Carlo settings
☒	☐	For hierarchical and complex designs, identification of the appropriate level for tests and full reporting of outcomes
☒	☐	Estimates of effect sizes (e.g. Cohen's *d*, Pearson's *r*), indicating how they were calculated

Our web collection on statistics for biologists contains articles on many of the points above.

Software and code

Policy information about availability of computer code

Data collection	No software was used for data collection.
Data analysis	We used the ImageJ software to quantify the protein bands intensity and used the GraphPad 7, Excel 16.30 to generate the graph figures and statistic analyses.

For manuscripts utilizing custom algorithms or software that are central to the research but not yet described in published literature, software must be made available to editors/reviewers. We strongly encourage code deposition in a community repository (e.g. GitHub). See the Nature Research guidelines for submitting code & software for further information.

Data

Policy information about availability of data

All manuscripts must include a data availability statement. This statement should provide the following information, where applicable:

- Accession codes, unique identifiers, or web links for publicly available datasets
- A list of figures that have associated raw data
- A description of any restrictions on data availability

All relevant data are included in the paper and supplementary information files. Uncropped images for immunoblots and statistical source data are provided with this paper. MS data was used to characterize PDK1 phosphorylation residues, the raw data was deposited in iProX/ProteomeXchange under the accession code IPX0003428000/PXD028167 for figure S1, http://proteomecentral.proteomexchange.org/cgi/GetDataset?ID=PXD028167. The public cBioPortal for Cancer Genomics database was used with the website link: https://www.cbioportal.org/.

Field-specific reporting

Please select the one below that is the best fit for your research. If you are not sure, read the appropriate sections before making your selection.

☒ Life sciences ☐ Behavioural & social sciences ☐ Ecological, evolutionary & environmental sciences

For a reference copy of the document with all sections, see nature.com/documents/nr-reporting-summary-flat.pdf

Life sciences study design

All studies must disclose on these points even when the disclosure is negative.

Sample size	No sample size calculations were performed. Sample size was determined according to our experience as well as literature reporting in terms of specific experiment. For xenografted mouse models, at least 5 mice were randomly chose for tumor growth study. Sample size for in vitro experiments were predetermined with sufficient replicates to validate experimental outcomes.
Data exclusions	No data was excluded from the experiments.
Replication	Multiple independent repeats were included for related experiments. Each experiment including mass spectrometry was performed for at least three times and obtained similar results. Animal-related experiments have been done once to compare the growth rate between groups.
Randomization	At least 5/group 6-8 week female nude mice were chosen as xenografted hosts. All these mice were randomly allocated into experimental groups.
Blinding	For cell-based experiments Western blotting, cell types were known when prepare the samples or start to treat cells at the beginning of experiments. Data measurement for western blot, cell number, colony formation and soft agar assays were blinded to different person who processed assay at the time. The mouse tumor formation, staining were also measured and analyzed by different person blindly.

Reporting for specific materials, systems and methods

We require information from authors about some types of materials, experimental systems and methods used in many studies. Here, indicate whether each material, system or method listed is relevant to your study. If you are not sure if a list item applies to your research, read the appropriate section before selecting a response.

Materials & experimental systems

n/a	Involved in the study
☐	☒ Antibodies
☐	☒ Eukaryotic cell lines
☒	☐ Palaeontology
☐	☒ Animals and other organisms
☒	☐ Human research participants
☒	☐ Clinical data

Methods

n/a	Involved in the study
☒	☐ ChIP-seq
☒	☐ Flow cytometry
☒	☐ MRI-based neuroimaging

Antibodies

Antibodies used

Antibodies were diluted in TBST buffer with 5% non-fat milk for western blot.

Anti-AKT Substrate (RxxpS/T) antibody (110B7E, Rabbit mAb #9614, 1:1000), anti-phospho-Ser473-AKT antibody (D9E, Rabbit mAb #4060,1:3000), anti-phospho-Thr308-AKT antibody (C31E5E, Rabbit mAb #2965, 1:1000), anti-AKT total antibody (C67E7, Rabbit mAb #4691,1:3000), anti-PDK1 antibody (D4Q4D, Rabbit mAb #13037, 1:1000), anti-phospho-Ser241-PDK1 antibody (Rabbit #3061, 1:3000), anti-AIF antibody (D39D2, Rabbit mAb #5318, 1:1000), anti-phospho-Ser9-GSK3b antibody (D85E12, Rabbit mAb #5558,1:3000), anti-GSK3b antibody (D5C5Z, Rabbit mAb #12456, 1:1000), anti-phospho-FOXO1 (Ser256) antibody (Rabbit #9461, 1:1000), anti-Myc antibody (9B11, Mouse mAb #2276, 1:1000), anti-GST antibody (91G1, Rabbit mAb #2625, 1:1000), anti-pS6K1 (Thr389) antibody (Rabbit #9205, 1:1000), anti-S6K1 antibody (49D7, Rabbit mAb #2708, 1:1000), anti-S6 antibody (5G10, Rabbit mAb #2217, 1:3000) and anti-pS240/244-S6 antibody (D68F8, Rabbit mAb #5364, 1:3000), 14-3-3r (D15B7, Rabbit mAb #5522, 1:1000) were obtained from Cell Signaling Technology.

Polyclonal anti-HA antibody (Y11, Mouse, #sc-805, 1:1000) were obtained from Santa Cruz.

Polyclonal anti-Flag antibody (Rabit F-2425, 1:1000), monoclonal anti-Flag antibody (M2, Mouse #F-3165, 1:3000), anti-Tubulin antibody (B5-1-2, Mouse #T-5168, 1:3000), anti-Vinculin antibody (VIN-11-5, Mouse #V4505, 1:3000), anti-Flag agarose beads (M2, Mouse A-2220), anti-HA agarose beads (A-2095), peroxidase-conjugated anti-mouse secondary antibody (A-4416) and peroxidase-conjugated anti-rabbit secondary antibody (A-4914) were obtained from Sigma. Anti-pS6K1 (Thr229) antibody (ab5231, 1:1000) was obtained from abcam.

Monoclonal anti-HA antibody (16B12, Mouse #901503, 1:3000), anti-GAPDH (FF26A/F9, mouse #649201, 1:3000) antibody was obtained from Biolegend.

The polyclonal phosphorylation antibodies against pS549-PDK1 (1:1000) generated by Abclonal Technology were derived from rabbit with four clones. The antigen sequence used for immunization was PDK1 aa544-552 (C-RQRYQSHPD).

Anti-Ki67 (SP6, Mouse #ab16667, 1:200), anti-pS240/244-S6 (D68F8, Rabbit mAb CST #5364, 1:500) were used for immunohistochemistry staining.

Validation

All antibodies used in our study have been validated and detailed information could be found on the website from manufactures as listed below. Some of them have also been validated by our experiments as shown in this manuscript using either over-express, knockout or knockdown strategies.

The polyclonal phosphorylation antibodies against pS549-PDK1 generated by Abclonal Technology were derived from rabbit with four clones. The antigen sequence used for immunization was PDK1 aa544-552 (C-RQRYQSHPD). This antibody was validated with dot blot assays and mutation western blot assays in Fig 1b-g, S3a-f.

Phospho-Akt Substrate (RXXS*/T*) antibody, https://www.cellsignal.com/products/primary-antibodies/phospho-akt-substrate-rxxs-t-110b7e-rabbit-mab/9614

phospho-Ser473-Akt, https://www.cellsignal.com/products/primary-antibodies/phospho-akt-ser473-d9e-xp-rabbit-mab/4060

phospho-Thr308-Akt, https://www.cellsignal.com/products/primary-antibodies/phospho-akt-thr308-c31e5e-rabbit-mab/2965?site-search-type=Products&N=4294956287&Ntt=%282965%29%2C&fromPage=plp&_requestid=740425

Akt1 antibody, https://www.cellsignal.com/products/primary-antibodies/akt-pan-c67e7-rabbit-mab/4691?site-search-type=Products&N=4294956287&Ntt=%284691%29%2C&fromPage=plp&_requestid=740463

Akt total antibody, https://www.cellsignal.com/products/primary-antibodies/akt1-c73h10-rabbit-mab/2938?site-search-type=Products&N=4294956287&Ntt=%282938%29%2C&fromPage=plp&_requestid=740451

pPDK1(S241) antibody, https://www.cellsignal.com/products/primary-antibodies/phospho-pdk1-ser241-antibody/3061?_=1643507849797&Ntt=(3061),&tahead=true

pS6K1 (Thr389) antibody, https://www.cellsignal.com/products/primary-antibodies/phospho-p70-s6-kinase-thr389-antibody/9205

p70 S6 antibody, https://www.cellsignal.com/products/primary-antibodies/p70-s6-kinase-49d7-rabbit-mab/2708?site-search-type=Products&N=4294956287&Ntt=%282708%29&fromPage=plp

phospho-Ser9-GSK3b antibody, https://www.cellsignal.com/products/primary-antibodies/phospho-gsk-3b-ser9-d85e12-xp-rabbit-mab/5558?site-search-type=Products&N=4294956287&Ntt=%285558%29%2C&fromPage=plp&_requestid=740494

GSK3b antibody, https://www.cellsignal.com/products/primary-antibodies/gsk-3b-d5c5z-xp-rabbit-mab/12456?site-search-type=Products&N=4294956287&Ntt=%2812456%29&fromPage=plp&_requestid=740502

phospho-FOXO1 (Thr24)/FOXO3A (Thr32) antibody, https://www.cellsignal.com/products/primary-antibodies/phospho-foxo1-thr24-foxo3a-thr32-antibody/9464?site-search-type=Products&N=4294956287&Ntt=+%289464%29&fromPage=plp&_requestid=740513

pS240/244-S6 antibody, https://www.cellsignal.com/products/primary-antibodies/phospho-s6-ribosomal-protein-ser240-244-d68f8-xp-rabbit-mab/5364

FOXO3A antibody, https://www.cellsignal.com/products/primary-antibodies/foxo3a-75d8-rabbit-mab/2497?site-search-type=Products&N=4294956287&Ntt=%282497%29&fromPage=plp&_requestid=740526

AIF antibody, https://www.cellsignal.com/products/primary-antibodies/aif-d39d2-xp-rabbit-mab/5318?_=1643507985130&Ntt=(5318)&tahead=true

Myc-tag antibody, https://www.cellsignal.com/products/primary-antibodies/myc-tag-9b11-mouse-mab/2276?site-search-type=Products&N=4294956287&Ntt=%282276%29&fromPage=plp

GST antibody, https://www.cellsignal.com/products/primary-antibodies/gst-91g1-rabbit-mab/2625?site-search-type=Products&N=4294956287&Ntt=%282625&fromPage=plp&_requestid=740541

S6 antibody, https://www.cellsignal.com/products/primary-antibodies/s6-ribosomal-protein-5g10-rabbit-mab/2217?site-search-type=Products&N=4294956287&Ntt=%282217%29&fromPage=plp&_requestid=920765

14-3-3r antibody, https://www.cellsignal.com/products/primary-antibodies/14-3-3-g-d15b7-rabbit-mab/5522?site-search-type=Products&N=4294956287&Ntt=14-3-3&fromPage=plp

polyclonal anti-HA antibody (sc-805), https://www.scbt.com/scbt/product/ha-probe-antibody-y-11?requestFrom=search

Polyclonal anti-Flag antibody (F-2425), https://www.sigmaaldrich.com/catalog/product/sigma/f7425?lang=en&region=US

monoclonal anti-Flag antibody (F-3165, clone M2), https://www.sigmaaldrich.com/catalog/product/sigma/f3165?lang=en&region=US

Tubulin antibody (T-5168), https://www.sigmaaldrich.com/catalog/product/sigma/t5168?lang=en&region=US

Flag agarose beads (A-2220), https://www.sigmaaldrich.com/catalog/product/sigma/a2220?lang=en&region=US

HA agarose beads (A-2095), https://www.sigmaaldrich.com/catalog/product/sigma/a2095?lang=en&region=US

peroxidase-conjugated anti-mouse secondary antibody (A-4416), https://www.sigmaaldrich.com/catalog/product/sigma/a4416?lang=en&region=US

peroxidase-conjugated anti-rabbit secondary antibody (A-4914), https://www.sigmaaldrich.com/catalog/product/sigma/a4914?lang=en&region=US

Monoclonal anti-HA antibody (MMS-101P), https://www.biolegend.com/en-us/products/purified-anti-ha-11-epitope-tag-antibody-11374

Eukaryotic cell lines

Policy information about cell lines

Cell line source(s)	HEK293, HEK293T and DLD1 cells were obtained from ATCC. , DLD1-PDK1-/- and wild-type DLD1 cells were kindly provided by Dr. Bert Vogelstein (Johns Hopkins University School of Medicine). These cells were maintained in DMEM medium supplemented with 10% FBS. Other cells were cultured in DMEM medium supplemented with 10% FBS. sgPDK1 HEK293 cell lines were generated via a CRISPR/CAS9 methods
Authentication	Cell lines were not authenticated.
Mycoplasma contamination	Yes. Cell lines used in this study were routinely tested to be negative for mycoplasma.
Commonly misidentified lines (See ICLAC register)	No.

Animals and other organisms

Policy information about studies involving animals; ARRIVE guidelines recommended for reporting animal research

Laboratory animals	NU/J nude female mice at 4-6 week old were purchased from Sun Yat-sen University mouse facility in Fig 2f-g, 6d-f. Mice were housed in specific pathogen-free facilities with 12 hrs dark/light cycle.
Wild animals	No wild animals involved in this study.
Field-collected samples	This study didn't involve samples collected from field.
Ethics oversight	All animal experiments were approved by the Institutional Animal Care & Use Committee (IACUC) at University of Sun Yat-sen.

Note that full information on the approval of the study protocol must also be provided in the manuscript.
